# Honokiol inhibits carotid artery atherosclerotic plaque formation by suppressing inflammation and oxidative stress

**DOI:** 10.18632/aging.103120

**Published:** 2020-05-04

**Authors:** Yuan Liu, Peng Cheng, An-Hua Wu

**Affiliations:** 1 Department of Neurosurgery, The First Hospital of China Medical University, Shenyang, Liaoning, China

**Keywords:** atherosclerosis, honokiol, inflammation, oxidative stress, NF-κB

## Abstract

Honokiol is a natural active compound extracted from Chinese herbal medicine, *Magnolia officinalis*. In this study, the role of honokiol in the development of carotid artery atherosclerotic lesions was evaluated in an ApoE-/- mouse model fed with a normal diet (ND) or a Western-type diet (WD) for ten weeks. After first two weeks, a perivascular collar was surgically placed on the right common carotid arteries of the mice. Then, WD-fed mice were intraperitoneally injected with honokiol (10 or 20 mg/kg) or administrated with 10 mg/kg atorvastatin calcium by gavage once a day for eight weeks. After that, the right common carotid arteries were excised for further experiments. The result showed that honokiol substantially inhibited the development of atherosclerotic lesions. Furthermore, honokiol downregulated the expression of pro-inflammatory markers, like tumor necrosis factor-α, interleukin (IL)-6, and IL-1β. Additionally, honokiol treatment decreased reactive oxygen species level and enhanced superoxide dismutase activity. Nitric oxide level, inducible nitric oxide synthase (iNOS) expression, and aberrant activation of nuclear factor-κB pathway were also significantly inhibited by honokiol treatment. Together, these findings suggest that honokiol protects against atherosclerotic plaque formation in carotid artery, and may be an effective drug candidate for the treatment of carotid artery atherosclerotic stenosis.

## INTRODUCTION

Atherosclerosis (AS), caused by abnormal lipid metabolism, is a disorder featured by the plaque formation in large and medium-sized arterial walls [1]. AS is the major pathological basis of chronic cardiovascular diseases such as myocardial infarction and stroke [2], and there is an increased prevalence of AS, which has seriously affected the health of aging population. Recent studies have suggested that AS is a form of inflammation mainly caused by inflamed vascular smooth muscle cells (VSMCs) [3, 4]. The activation, migration, and proliferation of VSMCs from the media to the intima of the arterial wall are necessary steps in the formation of atherosclerotic plaques [4]. Therefore, suppressing inflammation in VSMCs may be a potential strategy to inhibit AS progression. In addition, compelling evidence has demonstrated that vascular oxidative stress participates in the pathogenesis of AS [5, 6]. Besides that, more recent studies have suggested that mitochondria [7], endothelial dysfunction [8], and interplay between inflammation and immune cells [9, 10] are associated with AS progression. However, the complicated pathogenesis of AS has not been fully understood. Therefore, in-depth study of the pathogenic mechanism of AS and searching for effective treatment are needed.

Honokiol is a small-molecule polyphenol, extracted from the Chinese herbal medicine, *Magnolia officinalis*. Honokiol possesses various pharmacological activities, such as anti-inflammatory, anti-thrombotic, and anti-tumor activities [11]. A series of recent studies also show that honokiol may have anti-atherogenic potential through anti-platelet [12], lowering blood pressure [13], and hypoglycemic action [14]. A previous study has showed that honokiol inhibits tumor necrosis factor-α (TNF-α)-induced neutrophil adhesion and vascular cell adhesion molecule-1 (VCAM-1) expression in cerebral endothelial cells by downregulating nuclear factor-κB (NF-κB), an inflammatory transcription factor [15]. Moreover, honokiol inhibits TNF-α-induced migration of VSMCs by suppressing the activation of NF-κB signaling pathway [16]. Honokiol has also been reported to ameliorate endothelial dysfunction in atherosclerotic cells [17]. However, it is not yet clear whether honokiol could inhibit atherosclerotic lesions *in vivo*. Therefore, in this study, we employed an *in vivo* carotid artery atherosclerotic plaque model in ApoE-/- mice, and investigated the effect of honokiol on the formation of atherosclerotic plaque and its potential biological mechanisms. This study may help us to develop new strategies for the treatment of carotid artery atherosclerotic stenosis.

## RESULTS

### Honokiol alleviated the formation of carotid atherosclerotic plaque in ApoE-/- mice

**Figure 1:**
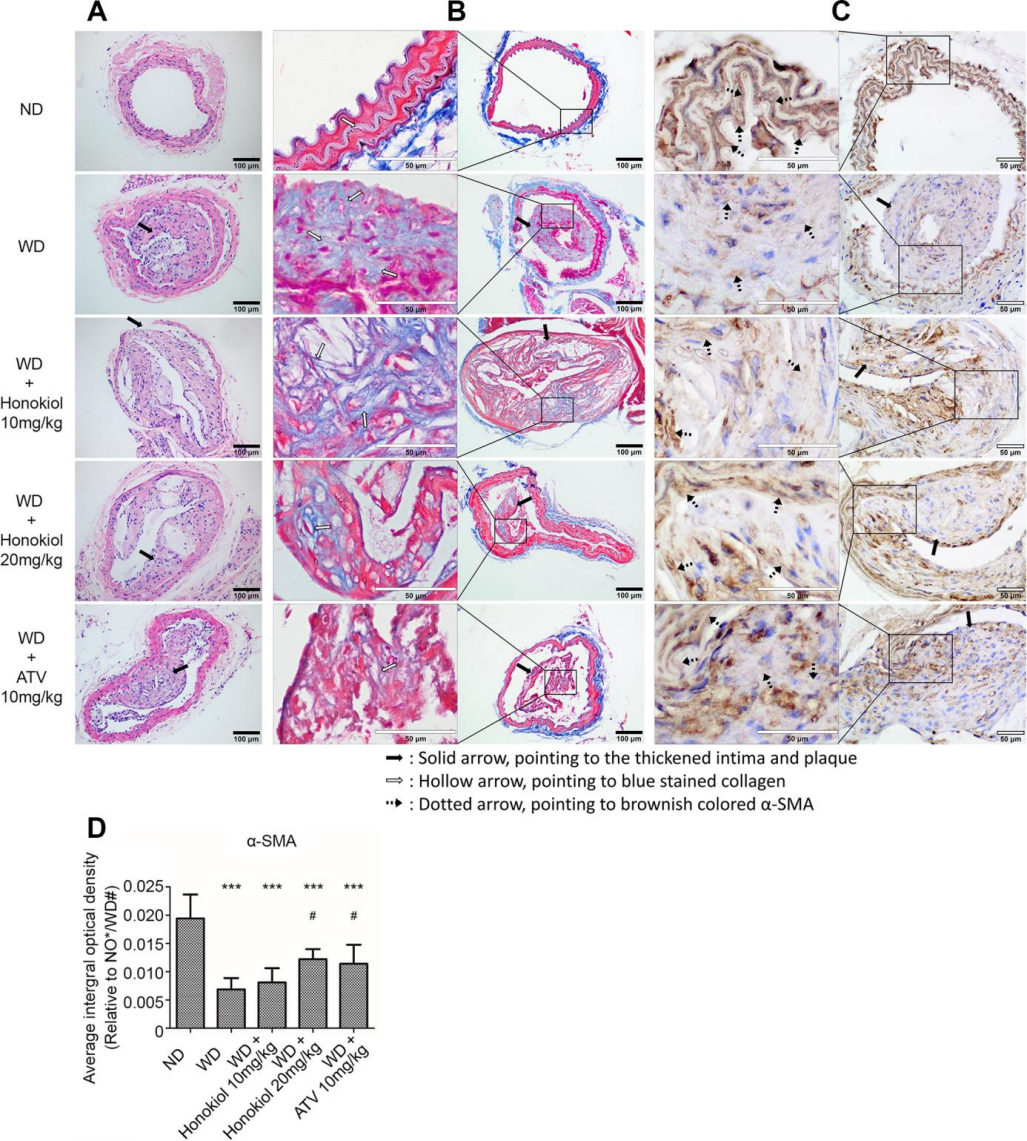
**Effect of honokiol on the formation of carotid atherosclerotic plaque in ApoE-/- mice.** (**A**) Representative HE staining images showing the formation of atherosclerotic plaque and vascular morphology changes (black solid scale bar: 100μm for 200×; black solid arrows point to the thickened intima and plaque). (**B**) Representative Masson trichrome staining images showing aortic collagen formation (black solid scale bar: 100μm for 200X; hollow scale bar: 50μm for 400X; black solid arrows point to the thickened intima and plaque; hollow arrows point to blue stained collagen). (**C**) The expression of α-SMA in carotid tissue was assessed by immunohistochemical staining (hollow scale bar: 50μm for 400X; black solid arrows point to the thickened intima and plaque; black dotted arrows point to brownish colored α-SMA). (**D**) The average integral optical density of α-SMA in carotid tissue was quantitatively analyzed by Gel-Pro Analyzer 4.5 software. (n = 6; *P < 0.05, **P < 0.01, ***P < 0.001, vs. the ND group. # *P* < 0.05, ## *P* < 0.01, ### *P* < 0.001, vs. the WD group; one-way ANOVA). ND: normal diet; WD: Western-type diet; α-SMA: α-smooth muscle actin.

To determine the effect of honokiol on the progression of atherosclerotic plaques, the sections of carotid arteries were subjected to HE staining. As shown in Figure 1A, the presence of atherosclerotic plaques and thickening of the artery walls were observed in the carotid artery of ApoE-/- mice fed with Western-type diet (WD). These changes could be alleviated by the treatment with honokiol or Atorvastatin calcium (ATV). Moreover, aortic collagen was detected by Masson trichrome staining. The result showed that the collagen deposition highlighted in blue by Masson trichrome staining was significantly enhanced in mice with AS (Figure 1B). As expected, honokiol and ATV treatment effectively inhibited collagen formation in arterial walls. Phenotypic switching of VSMCs by inhibiting the expression of α-smooth muscle actin (α-SMA) has been identified as one of the major causes of atherosclerotic plaque formation [19]. Thus, the formation of atherosclerotic plaque was further evaluated by immunohistochemical staining of α-SMA (Figure 1C, 1D). The data demonstrated that the expression of α-SMA was significantly decreased in the WD group. This also could be inhibited by honokiol or ATV treatment. Collectively, these data suggested that honokiol could alleviate the formation of carotid atherosclerotic plaque induced by WD *in vivo*.

### Honokiol inhibited the inflammatory response and oxidative stress in AS mice

**Figure 2:**
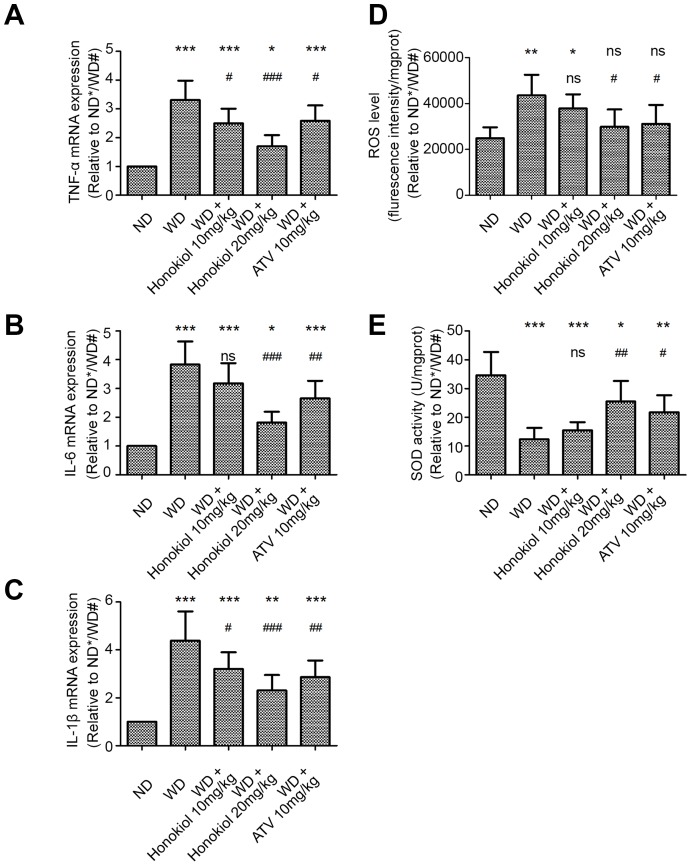
**Effect of honokiol on inflammatory response and oxidative stress in the carotid tissue of atherosclerotic mice.** (**A**–**C**) The mRNA expression of TNF-α (**A**), IL-6 (**B**), and IL-1β (**C**) in carotid tissue was detected by real-time PCR. (n = 6; * *P* < 0.05, ** *P* < 0.01, *** *P* < 0.001, vs. the ND group. # *P* < 0.05, ## *P* < 0.01, ### *P* < 0.001, vs. the WD group; one-way ANOVA). (**D**, **E**) The ROS level (**D**) and SOD activity (**E**) in carotid tissue were detected by commercial kits in the indicated group. (n = 6; * *P* < 0.05, ** *P* < 0.01, *** *P* < 0.001, vs. the ND group. # *P* < 0.05, ## *P* < 0.01, ### *P* < 0.001, vs. the WD group; one-way ANOVA). TNF-α: Tumor necrosis factor-α; interleukin-6: IL-6; and interleukin-1β: IL-1β; ND: normal diet; WD: Western-type diet; ROS: reactive oxygen species; SOD: superoxide dismutase.

Inflammatory response plays important roles in the occurrence and development of AS. To determine the effect of honokiol on inflammatory response, we measured the expression of three pro-inflammatory cytokines, including tumor necrosis factor (TNF)-α, interleukin (IL)-6, and IL-1β, in carotid tissue. As shown in Figure 2A–2C, compared with the normal diet (ND) group, the mRNA levels of TNF-α, IL-6, and IL-1β were significantly increased in the carotid tissues of ApoE-/- mice fed with WD. Treatment with honokiol or ATV significantly down-regulated the elevated expression of TNF-α, IL-6, and IL-1β induced by WD (Figure 2A–2C). Oxidative stress is triggered by inflammation during AS. Therefore, we further investigated the effect of honokiol on reactive oxygen species (ROS) production and superoxide dismutase (SOD) activity. As shown in Figure 2D, 2E, in comparison with the ND group, the level of ROS was increased, while the activity of SOD was decreased in the carotid tissues of AS mice. These changes could be reversed by honokiol or ATV treatment. In addition, there was a dose-dependent relationship with the therapeutic effect of honokiol, and 20 mg/kg honokiol had beneficial effects comparable to that of 10 mg/kg of ATV. Taken together, honokiol inhibited the inflammatory response and oxidative stress in AS mice.

### Honokiol suppressed nitric oxide (NO) production and inducible nitric oxide synthase (iNOS) expression in carotid tissue of AS mice

**Figure 3:**
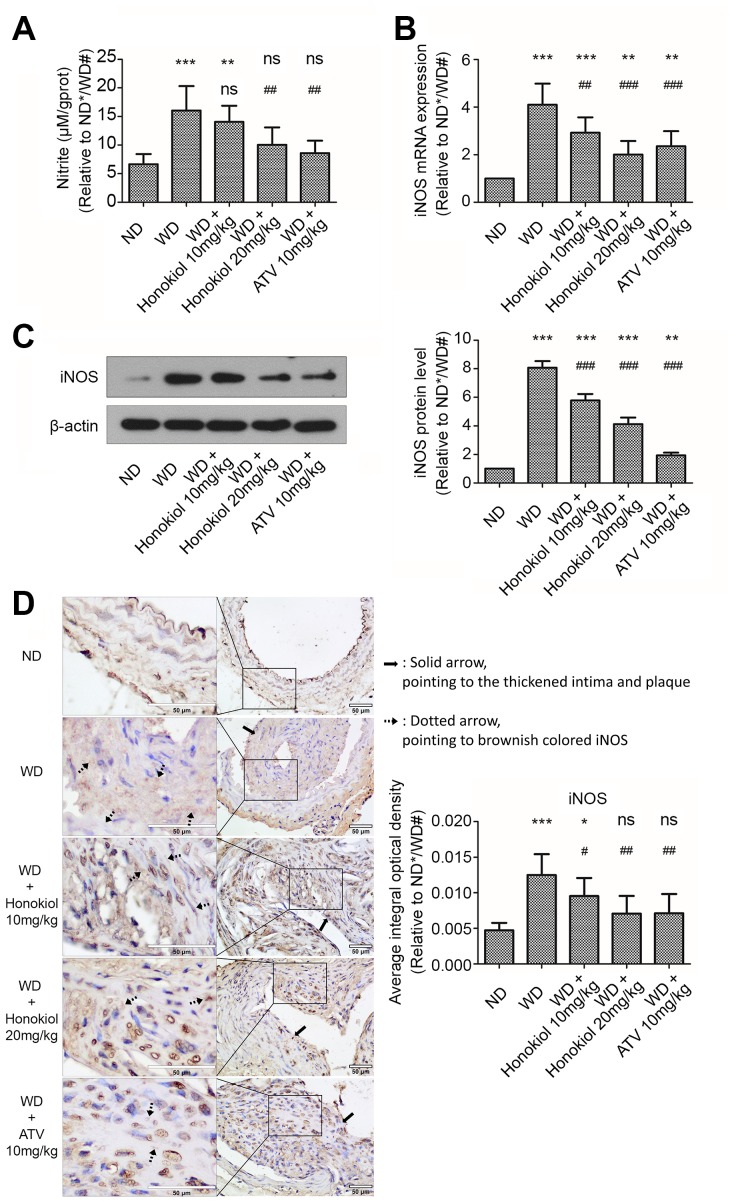
**Effect of honokiol on NO production and iNOS expression in the carotid tissue of atherosclerotic mice.** (**A**) The amount of nitric oxide in carotid tissue from the indicated experimental groups. (n = 6; * *P* < 0.05, ** *P* < 0.01, *** *P* < 0.001, vs. the ND group. # *P* < 0.05, ## *P* < 0.01, ### *P* < 0.001, vs. WD group; one-way ANOVA). (**B**) The mRNA expression of iNOS in carotid tissue obtained from the indicated groups was detected by real-time PCR. (n = 6; * *P* < 0.05, ** *P* < 0.01, *** *P* < 0.001, vs. the ND group. # *P* < 0.05, ## *P* < 0.01, ### *P* < 0.001, vs. the WD group; one-way ANOVA). (**C**) Western blotting was performed to evaluate iNOS protein expression in carotid tissue obtained from the indicated groups (upper panel). β-actin was served as a loading control. Quantification of the band density is shown in the right panel. (n = 6; * *P* < 0.05, ** *P* < 0.01, *** *P* < 0.001, vs. the ND group. # *P* < 0.05, ## *P* < 0.01, ### *P* < 0.001, vs. the WD group; one-way ANOVA). (**D**) Representative immunohistochemical staining images of iNOS in carotid tissue obtained from the indicated groups (hollow scale bar: 50μm for 400X; black solid arrows point to the thickened intima and plaque; black dotted arrows point to brownish colored α-SMA). The average integral optical density of iNOS in carotid tissue was quantitatively analyzed and shown in the right panel. (n = 6; * *P* < 0.05, ** *P* < 0.01, *** *P* < 0.001, vs. the ND group. # *P* < 0.05, ## *P* < 0.01, ### *P* < 0.001, vs. the WD group; one-way ANOVA). NO: Nitric oxide; iNOS: inducible nitric oxide synthase; ND: normal diet; WD: Western diet.

Next, we investigated whether honokiol influenced the production of NO, an important chemical messenger, in carotid tissue. As shown in Figure 3A, in comparison with the ND group, the level of NO was markedly upregulated in ApoE-/- mice fed with WD. This effect could be attenuated by honokiol or ATV treatment. Since the synthesis of NO is catalyzed by iNOS, we further investigated iNOS expression in carotid tissue. As shown in Figure 3B, iNOS mRNA expression was increased in mice with AS. As expected, the data confirmed that honokiol or ATV treatment could down-regulate the increased protein level of iNOS in carotid tissue of mice with AS (Figure 3C, 3D). In addition, the effect of honokiol was dose-dependent, and 20 mg/kg honokiol had stronger beneficial effects than 10 mg/kg of ATV. Together, honokiol suppressed NO production and iNOS expression in carotid tissue of AS mice.

### Honokiol inhibited the activation of NF-κB signaling pathway in AS mice

**Figure 4:**
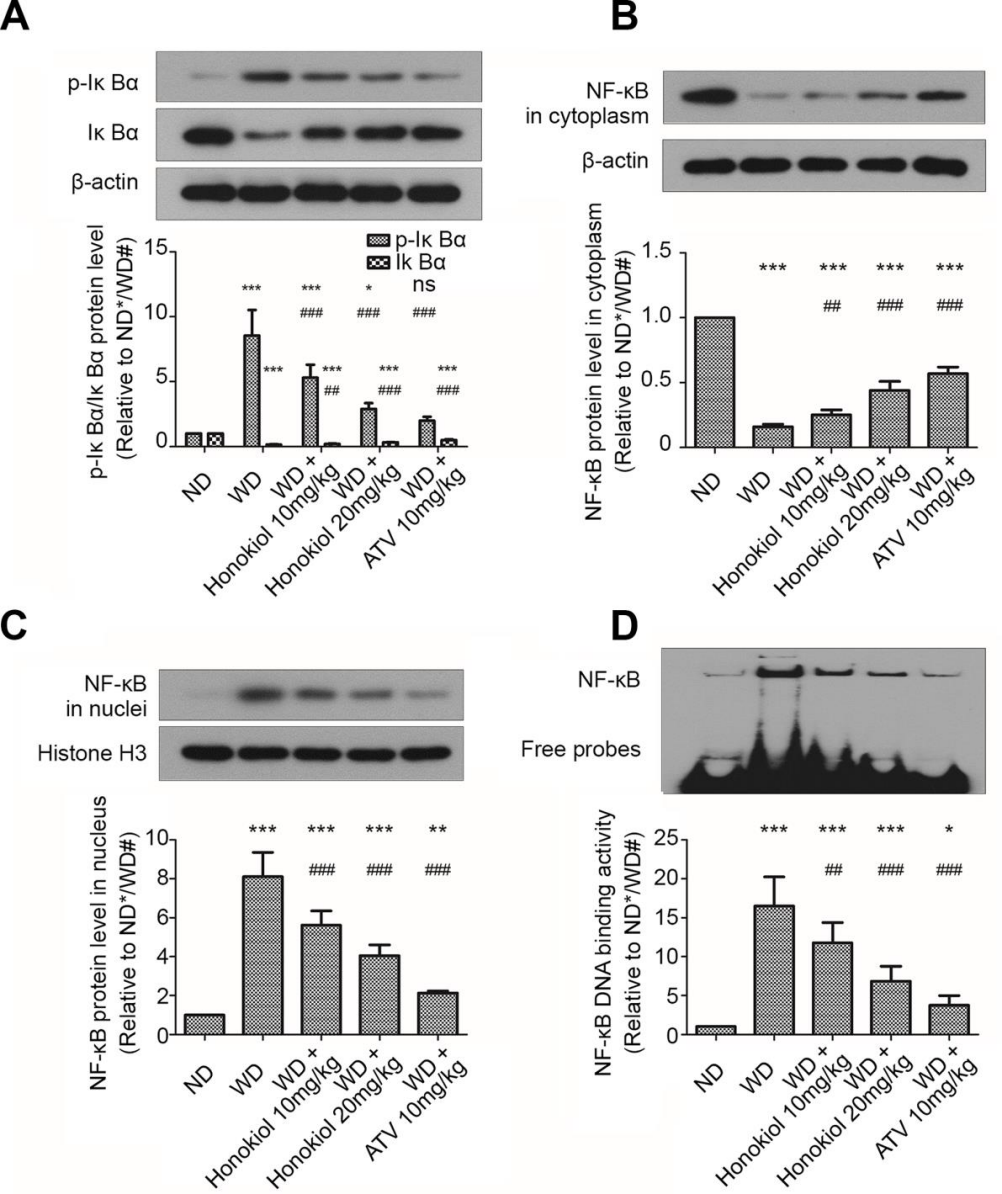
**Effect of honokiol on the activation of NF-κB signaling pathway in mice with carotid artery atherosclerosis.** (**A**–**C**) The protein levels of p-IκBα, IκBα (**A**), cytoplasmic NF-κB (**B**), and nuclear NF-κB (**C**) were assessed by western blotting analysis. β-actin or histone H3 was used as a loading control for cytoplasmic or nuclear protein, respectively. (n = 6; * *P* < 0.05, ** *P* < 0.01, *** *P* < 0.001, vs. the ND group. # *P* < 0.05, ## *P* < 0.01, ### *P* < 0.001, vs. the WD group; one-way ANOVA). (**D**) The DNA binding activities of NF-κB in carotid tissue from the indicated groups were detected by EMSA assay. (n = 6; * *P* < 0.05, ** *P* < 0.01, *** *P* < 0.001, vs. the ND group. # *P* < 0.05, ## *P* < 0.01, ### *P* < 0.001, vs. the WD group; one-way ANOVA). NF-κB: nuclear factor-κB; IκBα: inhibitor of NF-κB; ND: normal diet; WD: Western diet; EMSA: electrophoretic mobility shift assay.

The NF-κB signaling pathway has been reported to be involved in the regulation of inflammatory response [20]. Based on this observation, we sought to examine the effect of honokiol on NF-κB signaling pathway. As shown in Figure 4A–4C, compared with the ND group, WD group had an increased p-IκBα and NF-κB expression in the nuclei. Meanwhile, the expression levels of IκBα and NF-κB in the cytoplasm were reduced in ApoE-/- mice fed with WD. These changes could be suppressed by administration with honokiol or ATV (Figure 4A–4C). Moreover, the EMSA assay was performed to assess the DNA-binding activity of NF-κB. As shown in Figure 4D, the DNA-binding activity of NF-κB was enhanced in mice with AS. This effect could be attenuated by honokiol treatment. The effect of honokiol was still dose-dependent, but 20 mg/kg honokiol had less beneficial effects than 10 mg/kg of ATV. Together, honokiol had a protective effect against the activation of NF-κB signaling pathway in AS mice.

## DISCUSSION

As the recommendation of clinical guidelines in many countries [18], the effects of statins on the prevention of atherosclerotic cardiovascular and cerebrovascular accidents have been confirmed by a variety of studies [19–21]. However, statins related complications in clinical, such as rhabdomyolysis, new-onset diabetes mellitus and hemorrhagic stroke are dose-dependent [22]. Thus, new drug candidates to replace statins or at least reduce its dosage need to be further investigated.

The occurrence of AS is a complex pathological process that involves a variety of inducing factors. Honokiol has been demonstrated to have broad pharmacological activities [11]. In this study, we provided evidences on the anti-atherosclerosis effect of honokiol with an ApoE-/- mouse model and compare its effect with statins for the first time. ApoE-/- mice were first created in 1992, and may spontaneously develop hypercholesterolemia and AS [23, 24]. The pathological course, histopathologic distribution, and structure of ApoE-/- mice are very similar to those in humans; thus, ApoE-/- mouse is an ideal model for AS research [25]. According to our data, honokiol significantly suppressed the formation of atherosclerotic plaque in ApoE-/- mouse fed with WD. In mechanism, honokiol inhibited the inflammatory response and oxidative stress in AS mice by suppressing the activation of NF-κB signaling pathway and decreasing nitric oxide (NO) production.

In comparison to previous reports via cell models [15–17], the present work is an *in vivo* model study. Compared with the only published study of honokiol on its antiatherogenic effects *in vivo* which used carotid artery balloon injury model with perivascular honokiol application [26], we employed the WD-induced arteriosclerosis model with intraperitoneal injection of honokiol. Since our modeling method did not directly damage the integrity of vascular intima, the atherosclerotic vessels obtained were more in line with the real pathophysiological process of atherosclerotic development. Besides, the pharmacokinetics study indicated that part of the pharmacological activity of honokiol might be generated by its conjugated metabolites [27]. Therefore, the clinical relevance of current data, obtained by intraperitoneal administration of honokiol, is closer to the actual clinical situation.

Growing evidence has indicated that AS is a chronic inflammatory disease [1]. Inflammation and a variety of pro-inflammatory cytokines play important roles in the occurrence and development of AS. TNF-α is an important pro-inflammatory cytokine expressed in atherosclerotic lesions [28]. The level of TNF-α is closely related to carotid intima-media layer thickening, dyslipidemia, and AS [29]. Ohta et al. showed that knockdown of TNF-α expression significantly decreased the atherosclerotic plaque area in ApoE-/- mice [30]. IL-6 is another representative inflammatory cytokine and its expression is significantly elevated in local plaques of AS [31]. Furthermore, it has been confirmed that IL-6 is a risk factor for AS, which is comparable to blood pressure and cholesterol level [32]. An injection of recombinant IL-6 can promote atherosclerotic plaque formation in ApoE-/- mice [33]. Moreover, an anti-mouse IL-6 receptor antibody (MR16-1) could suppress the formation of atherosclerotic lesion in LDLr-/- mice [34]. IL-1β has also been proved to contribute to the progression of AS [35]. Bhaskar et al. showed that XOMA 052, an anti-IL-1β antibody, inhibited the secretion of atherogenic cytokines and the release of degradative enzymes *in vitro*, and suppressed atherosclerotic lesion formation *in vivo* [36]. Our results showed that honokiol treatment effectively inhibited the expression of TNF-α, IL-6, and IL-1β in carotid tissue of ApoE-/- mice fed with WD, even better than ATV. This suggested that honokiol might be a potential drug for the treatment of AS. Furthermore, oxidative stress is considered to be involved in the pathological processes of AS. ROS production, due to an oxidative and anti-oxidative imbalance, may cause endothelial cell damage and monocyte activation to macrophages [37]. Moreover, excessive generation of ROS can induce the proliferation and migration of VSMCs, which aggravates intimal hyperplasia [38]. Therefore, excessive ROS production promotes AS progression. Our results showed that the ROS level in AS mice was markedly downregulated by honokiol. This also indicated a promising future for honokiol in the development of therapeutic strategy for AS. SOD is a native antioxidant enzyme that can eliminate ROS and protect the body from oxidative damage. It has been reported that enhancing SOD activity may delay the development of AS [5]. In the present study, the activity of SOD in the carotid tissue was enhanced by treatment with honokiol. This may be one of the mechanisms for the anti-AS activity of honokiol.

NO, is an important bioactive molecule and widely exists in various cells and tissues. Under physiological condition, NO has a variety of functions, including mediating vascular smooth muscle relaxation, preventing platelet aggregation, and reducing pulmonary circulation resistance [39]. However, under the pathological condition, excessive production of NO may cause tissue damage [40]. In recent years, the role of NO in the formation of AS has aroused extensive attention of researchers. It has been shown that iNOS-mediated NO production plays a key role in AS progression [41]. Knockout of iNOS significantly decreased lipid peroxidation level in serum and the atherosclerotic lesion area in ApoE-/- mice [42]. Our results demonstrated that the increased NO production and iNOS expression during AS progression were significantly inhibited following the administration of honokiol. This may be another mechanism for the anti-AS activity of honokiol.

It has been reported that NF-κB as a transcription factor regulates the expression of various inflammation-related genes. Under normal conditions, NF-κB binds to its inhibitory protein, IκBα, and exists in the cytoplasm in an inactive state. However, in response to inflammatory stimuli, NF-κB may translocate into the nucleus and bind to the promoters of its target genes to mediate their expression. A previous study has shown that NF-κB played a crucial role in regulating the expression of inflammatory mediators during AS [43]. Recent data have also demonstrated that NF-κB had a major impact on all stages of plaque formation [44]. Therefore, specific inhibitors of NF-κB may provide new therapeutic strategies to control the chronic inflammatory process of AS. According to a previous study, honokiol inhibited the activation of NF-κB pathway via suppressing pentraxin3 expression in an atherosclerotic cell model [17]. Furthermore, honokiol was demonstrated to restrain TNF-α-mediated NF-κB activation via repressing IκBα degradation and IκB kinase activation in rat aortic smooth muscle cells [16]. Consistent with these observations, the present study demonstrated that honokiol treatment effectively inhibited NF-κB pathway activation through enhancing IκBα expression and suppressing the phosphorylation of IκBα. Therefore, honokiol may relieve atherosclerosis by blocking NF-κB pathway in AS mice. However, whether suppression of PTX3 expression is implicated in honokiol-mediated NF-κB inhibition needs to be investigated in the future.

In summary, our findings suggest that honokiol could significantly suppress the formation of carotid artery atherosclerotic plaque in AS mice. Inhibition of the inflammatory response, oxidative stress, excessive production of NO, and the activation of NF-κB signaling pathway contributes to the protective effect of honokiol against AS. we provide evidence that honokiol may be a potential drug for preventing the formation and development of AS.

## MATERIALS AND METHODS

### Drugs

Honokiol was obtained from Aladdin Bio-science and Technology Co., Ltd (Shanghai, China) and dissolved in corn oil. The purity of honokiol was more than 98% as assessed by High Performance Liquid Chromatography. The molecular formula of honokiol is C18H18O2 and the molecular weight is 266.33. Atorvastatin calcium (ATV) was purchased from Meilun Biotech Co., Ltd (Dalian, China) and dissolved in saline before use.

### Animals and experimental protocol

Male ApoE-/- mice (six weeks old) were purchased from Vital River Laboratories Co., Ltd. (Beijing, China). The mice were fed with ND or WD (containing 0.25% cholesterol and 15% cacao butter) for ten weeks. Carotid artery plaque formation was induced by surgical placement of a perivascular collar on the right common carotid arteries according to a previously published method after WD feeding for two weeks [45]. Briefly, the mice were anaesthetized by intraperitoneal injection of 10% chloral hydrate (3 ml/kg). The right common carotid artery was exposed, and then a disinfectant collar (length, 3 mm; diameter, 0.3 mm) was placed and fixed around the right common carotid artery. The mice in the ND group underwent the same procedure except for collar placement. The WD-fed mice were randomly divided into four groups, which were treated with an intraperitoneal injection of vehicle (0.02 ml corn oil), 10 mg/kg honokiol, 20 mg/kg honokiol, or oral gavage of 10 mg/kg ATV once a day for eight weeks, respectively. The given dosage and duration of honokiol was under the reported safe dose [46], in which there was no significant accumulation or clinical parameters increasing in mice treated with 6.5mg magnolol and 2mg honokiol daily for less than 3 months. The ND-fed mice were intraperitoneally injected with vehicle (0.02 ml corn oil) to serve as a control. Eight weeks after the surgery, the mice were sacrificed by intraperitoneal injection of pentobarbital sodium (100 mg/kg). The right common carotid arteries were excised, and then fixed in 10% formaldehyde or quickly frozen in liquid nitrogen for further tests. All animal experiments were approved by the Medical Ethics Committee of China Medical University.

### Hematoxylin-eosin (HE) and Masson trichrome staining

The fixed carotid artery tissues were dehydrated, embedded in paraffin wax, sliced into 5-μm sections, and mounted on glass slides. Routine HE and Masson trichrome staining were performed according to standard histopathological methods. Vascular morphological changes and atherosclerotic plaque formation were observed by HE staining. Collagen formation in carotid arteries was assessed by Masson trichrome staining. The sections were photographed under a microscope (Olympus, Japan).

### Immunohistochemical staining

The expression of α-smooth muscle actin (α-SMA) and inducible nitric oxide synthase (iNOS) in carotid artery tissues was determined by immunohistochemical staining. In brief, 5-μm paraffin sections were immersed in antigen retrieval solution and heated for 10 min in a microwave oven. Then, the sections were then incubated with 3% H_2_O_2_ for 15 min at room temperature. After blocking with goat serum (Solarbio, Beijing, China) for 15 min at room temperature, the sections were incubated with primary antibodies separately at 4°C overnight (anti-α-SMA, 1:200, BOSTER, Wuhan, China; or anti-iNOS, 1:200, Proteintech, Wuhan, China). Biotinylated Goat anti-Mouse or Goat anti-Rabbit IgG (1:200, Beyotime, Haimen, China) were then added to the sections for 30 min at 37°C. After development with DAB and counterstaining with hematoxylin, the sections were photographed by a light microscopy.

### Detection of total nitric oxide (NO) in carotid tissue

Total NO level (μmol/g prot) was measured by a commercial NO Assay kit (Nanjing Jiancheng Bioengineering Institute, China), according to the manufacturer’s instructions.

### Detection of ROS and SOD activity in carotid tissue

The level of ROS and SOD activity in carotid tissue were determined by a ROS Assay Kit (Nanjing Jiancheng Bioengineering Institute, China) and a SOD Assay Kit (Nanjing Jiancheng Bioengineering Institute, China), respectively. The ROS level was shown by fluorescence intensity and the SOD activity was expressed as “U” of SOD/mg prot.

### Real-time polymerase chain reaction (PCR) analysis

The mRNA expression in carotid tissue was detected by real-time PCR. Briefly, total RNA was isolated from carotid tissue using the RNApure extraction kit (BioTeke Corporation, Beijing, China) following the manufacturer’s instructions. The RNA was reversely transcribed to first-strand DNA using Super M-MLV reverse transcriptase (BioTeke Corporation, China). The PCR experiment was performed using the SYBR Green method on the ExicyclerTM 96 Real-Time Quantitative Thermal Block (Bioneer Corporation, Korea). The primer sequences are listed in Table 1. The results were calculated using the 2^-ΔΔCt^ method and β-actin was used as a control.

**Table 1:** Primer sequences used for real-time PCR.

**Name**	**Sequence (5'-3')**	**Length**
TNF-α F	AGAAAGCATGATCCGCGAC	19
TNF-α R	TTGTGAGTGTGAGGGTCTGG	20
IL-1β F	TTGGGCCTCAAAGGAAAGAAT	21
IL-1β R	TGCTTGTGAGGTGCTGATGTA	21
IL-6 F	GCCCACCAAGAACGATAGTCAA	22
IL-6 R	CATTTCCACGATTTCCCAGA	20
iNOS F	GCAGGGAATCTTGGAGCGAGTTG	23
iNOS R	GTAGGTGAGGGCTTGGCTGAGTG	23
β-actin F	CTGTGCCCATCTACGAGGGCTAT	23
β-actin R	TTTGATGTCACGCACGATTTCC	22

### Western blotting analysis

The protein sample of carotid tissue was prepared with RIPA lysis buffer (Beyotime, China). The cytoplasmic and nuclear protein was extracted with a Nuclear and Cytoplasmic Protein Extraction Kit (Beyotime, China). The protein concentration was determined with a BCA Protein Assay Kit (Beyotime, China). Forty μg protein of each sample was separated by sodium dodecyl sulfate polyacrylamide gel electrophoresis (SDS-PAGE), and then transferred to PVDF membranes. After blocking with 5% skim milk for 1 h, the primary antibodies of iNOS (Sangon Biotech, China, 1:1000), inhibitor of nuclear factor kappa-B (IκBα) (Bioss, China, 1:500), phosphorylated (p)-IκBα (Bioss, China, 1:500), NF-κB (Sangon Biotech, China, 1:1000), and β-actin (Bioss, China, 1:500) were added to the membranes, respectively. Then the membranes were incubated at 4°C overnight. Then the HRP-conjugated secondary antibody (Beyotime, China, 1:5000) was added. The bands were visualized by BeyoECL Plus reagent (Beyotime, China) and the band densities were quantitatively analyzed by Gel-Pro Analyzer 4.5 software (Media Cybernetics, Bethesda, USA).

### Electrophoretic mobility shift (EMSA) assay

The EMSA assay was performed with a NF-κB EMSA kit (Viagene, China). In brief, the nuclear protein in carotid tissue was isolated as mentioned above. After assessment of the protein concentration by the BCA method, the samples were mixed with a labeled NF-κB probe and loaded onto a 6.5% non-denaturing gel. The electrophoresis was performed at 180 V for 80 min. The proteins were then transferred to a nylon membrane, crosslinked under UV radiation for 30 min, and detected by chemiluminescence.

### Statistical analysis

All statistical analyses were performed with GraphPad Prism 6 software. The experimental data are presented as means ± standard deviation (SD). The differences among experimental groups were determined by one-way analysis of variance (ANOVA) followed by Newman-Keuls Multiple Comparison Test. A statistically significant difference was identified when the *P* value was less than 0.05. All experiments results shown are representative of at least three independent experiments.

### Ethics approval and consent to participate

This study was approved by the Medical Ethics Committee of the First Hospital of China Medical University.

### ORCID

Yuan Liu https://orcid.org/0000-0001-7085-0113
